# Joint Cognition: Thought Contagion and the Consequences of Cooperation when Sharing the Task of Random Sequence Generation

**DOI:** 10.1371/journal.pone.0151306

**Published:** 2016-03-15

**Authors:** John Nicholas Towse, Andrea Sarah Towse, Satoru Saito, Yukio Maehara, Akira Miyake

**Affiliations:** 1 Department of Psychology, Lancaster University, Lancaster, United Kingdom; 2 Graduate School of Education, Kyoto University, Kyoto, Japan; 3 Faculty of Education, Nagasaki University, Nagasaki, Japan; 4 Department of Psychology and Neuroscience, University of Colorado Boulder, Boulder, Colorado, United States of America; University College London, UNITED KINGDOM

## Abstract

Generating random number sequences is a popular psychological task often used to measure executive functioning. We explore random generation under “joint cognition” instructions; pairs of participants take turns to compile a shared response sequence. Across three studies, we point to six key findings from this novel format. First, there are both costs and benefits from group performance. Second, repetition avoidance occurs in dyadic as well as individual production settings. Third, individuals modify their choices in a dyadic situation such that the pair becomes the unit of psychological function. Fourth, there is immediate contagion of sequence stereotypy amongst the pairs (i.e., each contributor “owns” their partner’s response). Fifth, dyad effects occur even when participants know their partner is not interacting with them (Experiment 2). Sixth, ironically, directing participants’ efforts away from their shared task responsibility can actually benefit conjoint performance (Experiment 3). These results both constrain models of random generation and illuminate processes of joint cognition.

## Introduction

Cognitive psychology relies overwhelmingly on what individuals think and do. That is, cognitive psychologists almost always test individuals and analyze their behaviors in isolation to investigate cognitive processes and architectures. Even when individuals are studied in social environments as a group, the individual responses are usually the only available metric of analysis. Yet even brief reflection confirms that in the real world much personal and professional decision-making involves, as a minimum, consultation with others, and more often extensive collaborative interactions. These situations lead to shared decisions amongst group members. Whilst there is a growing recognition of the relevance of social factors in shaping basic cognitive processes such as working memory (see for example [1]), there is a need for studies that identify how group performance compares with individual performance for popular cognitive paradigms and constructs. In the present article we draw on the key domain of executive function (EF).

EF is a broad term that refers to the deliberate, volitional, and purposeful control of behavior towards target outcomes. EF involves multiple dimensions or constraints that modulate mental activity so as to be more effective and appropriate (e.g., [2–3]). Given the centrality of this psychological concept in cognitive psychology, it is important to understand how behavioral regulation is affected by a group setting. We need to do so first, to understand better this cognitive construct of EF in its own right, and second, to understand the cognitive dynamics of group performance.

For several reasons, random number generation is a valuable paradigm to explore the construct of EF. The task is generally recognized to draw upon multiple, core, executive processes including the inhibition of stereotypical sequences and the planning and monitoring of output [3–4]. It has been deployed as a primary and secondary task [5] as well as being used in large-scale individual differences (latent variable) analyses [6]. Moreover, random generation has also been used to illuminate dysfunctional psychological states [7]. Thus, it has been a well studied paradigm that has been shown to be highly sensitive to different types of experimental and interindividual variations.

In this paper, we adopt the term “joint cognition”, to refer to a situation where a pair of individuals contribute to a shared cognitive goal. The term is explicitly designed to be distinguishable from, but resonate with, “joint action” (e.g. [8–9]). In joint action research, group members are usually assigned different sub-tasks with respect to a coordinated common purpose (e.g. in a motion task, one person accelerates and another brakes), and the emphasis is on response actions. Our focus here also centers on shared contributions to a task, but is oriented towards understanding the internal cognitive mechanisms involved, and each person has the same choices. The current term is clearly allied also to the phrase “collaborative cognition” sometimes used in the literature. However, for reasons we will discuss more explicitly later, we wish to remain neutral on the extent to which explicit collaboration is involved in shaping responses. Potentially in joint cognition, individuals may respond irregularly (e.g., brainstorming, essentially when ideas come to people they produce them ad libitum) or regularly (e.g., when contributions occur via a regular pattern such as alternation between response producers, as is the case with the current study). We will focus here on joint cognition in pairs following regular production sequences.

The choice of random number generation, which involves the production of a set of responses so as to form as random a sequence as possible, as a paradigm to explore joint cognition is driven by several considerations. First, the repeated, rapid selection from among a closed set of known response alternatives allows for response duties to be straightforwardly shared or rotated, effectively preventing undue influence from one member of the pair who dominates output. Second, randomization performance is partly assessed by looking for detectable relationships between discrete choices; what is relevant is the compound of many selections, not just one on its own. This also distributes responsibility across the pair and across time (there is not a single answer that terminates the task or solves the problem). Finally, random number generation usually involves responding to a rhythmic timing signal; this temporal consistency minimizes the biasing impact of “production blocking” (the putative impairment in collaborative situations from waiting and thus stalling responses [10]).

From one perspective, pairs should outperform individuals because each pair possesses (on average) twice the mental resources available to an individual. Insofar as random generation is argued to be a resource-constrained task [11], increasing the pool of resources should lead to an improvement in performance. Whether this resource advantage can be effectively deployed, however, is a key empirical question. Indeed, other research suggests that group activity often leads to productivity loss (i.e., suboptimal performance), compromising for example collaborative memory recall, problem solving, and brainstorming (see [12–15]). This type of research predicts that joint cognition will be inferior to that from individuals alone. However, both of these opposing predictions are essentially global perspectives (i.e., “groups will do better” vs. “groups will do worse”). Since random generation actually involves multiple components, reflecting the subtlety and complexity of many real life situations and dilemmas, we next articulate specific hypotheses for how joint cognition might influence particular aspects of random generation performance.

First, a major task demand in random number generation lies in the regulation and inhibition of prepotent or automatic sequence associations [11]. As such, the inhibition of “runs” of neighboring values (e.g., “4…5…6”) along with idiosyncratic number pair associations is highly sensitive to variables such as response speed [4] and concurrent working memory demands [16]. Since each member of a pair may have different sets of preferred associations (i.e., idiosyncratic chunks), we predict that collaborative sequences will contain fewer overall stereotyped patterns because patterns will be broken by turn-taking. At the same time, we also predict that associate responses from numerical neighbors (e.g., 5 followed by 6, or 3 followed by 2) will still be prevalent since these are shared, overlearned sequences for all participants. These predictions can be evaluated with reference to popular “Digram Use” and “Adjacency” measures of neighboring response values, respectively (all the performance scores are defined and explained in detail below).

Second, a widespread performance limitation relates to response repetitions. Ideally, repeating response values should occur as frequently as any other specific non-repeating alternative. Yet, among children and adult sequences, it is actually the least popular paired choice [17–18]. One theoretical account of repetition avoidance is that individuals self-suppress just produced answers, thought to be a general cognitive mechanism that avoids continuous perseveration on a recently activated mental representation [19]. For example, competitive queuing models within connectionist networks [20] instantiate this phenomenon to explain language production and serial order memory phenomena. These perspectives predict more repetitions and shorter repetition gaps in joint sequences, since alternate responses come from someone else. Essentially, even if one self-suppresses one’s own choices, that leaves a partner’s choice available for selection. On the other hand, if participants fully embody their partner’s contribution within the production task, then repetition avoidance will persist across experimental conditions. In contrast to standard views in the literature, this alternative view would thereby suggest that repetition avoidance is not just an output effect. We use an “Immediate Repetitions” score to evaluate this issue.

Third, random generation is limited by the ability to produce an even distribution of response choices. Over the full sequence, all response alternatives ought to be considered without preference, and this requirement is captured by the concept of the “equipotentiality criterion” [4]. In fact, the demands of concurrently and actively representing all choices explains why random number generation is easier when the response set is visible, thereby reducing the maintenance load on working memory [16] and why random number generation is harder when response sets are large and more responses must be tagged and monitored [4]. This constraint also helps explain why random number generation is harder than random keypress generation [4] since the number task requires an internal representation of responses. However, since many individuals cope reasonably with this equipotentiality criterion under normal response conditions and moderate-size response sets (i.e., only ten possible response alternatives), we do not predict strong effects of joint cognition on this aspect of random number generation. This “even distribution” aspect of random generation performance can be tracked with a “Redundancy” score.

To preview the empirical work to follow, there are three joint cognition studies. In Experiment 1, we study individuals alone and also bring them into pairs, comparing performance with respect to individual and group response rates, and analyze group performance as well as the individual contribution to that group. In Experiment 2, individuals take turns to contribute alongside an experimenter reading from a script prepared in advance, so as to determine whether dyadic interaction is important for the phenomena we have established. In Experiment 3, we compare instructions to groups to cooperate with each other against instructions to ignore partners. This allows us to consider whether cooperative intent is responsible for specific features of group performance.

## Experiment 1

To compare conjoint and individual performance, we administered the random generation task three times. Each participant produced sequences (1) alone at a slow pace (3 seconds per digit), (2) alone at a fast pace (1.5 seconds per digit), and (3) within a dyadic pair. In the last dyadic-pair condition, the overall pace for the pair was fast (1.5 seconds per digit), meaning that an individual member of the pair contributed to this turn-taking sequence at a slow pace for them (3 seconds per digit). Accordingly, the two solo conditions provided separate comparison points for the joint sequence, as well as providing baselines for interpreting each individual’s contribution to the paired sequence. All participants also completed a self-report measure of Social Desirability [21]. The purpose was to evaluate whether individual orientation towards or away from social alignment would be linked to joint cognition performance, for example with respect to reactivity.

To summarize and preview, this initial study addresses several research goals. We investigate whether joint cognition requirements produce productivity loss or gain for several executive control dimensions, as captured by a random generation task. This permits assessment of whether participants either react to or ignore their partner’s decisions. In addition, group performance offers the opportunity to evaluate hypotheses such as the self-suppression explanation for repetition avoidance.

### Method

#### Participants

Forty undergraduates from the University of Colorado Boulder and Lancaster University participated for partial fulfillment of a course requirement or for payment (there were no significant effects of participant source). Only one pair of participants knew each other prior to the experimental session, but their data are included in the analysis because their performance was within 1 SD of the group means. Ages were not recorded; data were collected in 2007.

This experiment was conducted in accordance with the Declaration of Helsinki and the Ethical Principles of the British Psychological Society (BPS) and the American Psychological Association (APA). The study was approved by the relevant ethics committees at the University of Colorado Boulder and at Lancaster University. All participants read and signed a consent form before participation.

#### Procedure

Initially, all participants completed the Social Desirability questionnaire. Afterwards they completed three random number generation tasks; two were performed individually and one as a pair. There were two experimenters and consequently individual performance was assessed simultaneously for each member of the pair. The order of task presentation was counterbalanced (individual or paired condition first, and within the former, the slow or fast sequence first).

Instructions followed a common format used for random number generation studies. They emphasized that participants should produce as random a sequence as possible, and the instructions included reference to the need to select all responses equally often and avoid sequence patterns. Participants were encouraged to imagine repeatedly rolling a many sided die and reporting the numbers this produced. For individual sequences, participants generated 100 numbers between 1 and 10 inclusively, one response every 1.5 seconds (fast condition) and one response every 3 seconds (slow condition). A metronome provided a tone at the appropriate rate and participants produced a number on or immediately after this signal. The importance of maintaining response pace was made explicit (actual missed time signals were recorded, but were very infrequent). The full instruction texts are available as part of the data deposit.

In the paired condition, participants took it in turns again to generate a number between one and ten every 1.5 seconds (therefore each participant contributed one response every 3 seconds). Participant pairs were instructed to make the *combined* sequence as random as possible. Each person generated 100 numbers, resulting in a paired sequence of 200 numbers.

### Results

Random sequence generation produces a rich data set that can be assessed in many different ways, but measures can be categorized into several major clusters. Therefore, we focus on a subset of indices representative of these clusters that are relevant to our research questions ([22] provide full computational details of the measures). For ease of interpretation, we report all analyses by stating the condition in which sequences were more (or less) random, rather than stating numeric values themselves, since some metrics involve in effect reverse scoring.

#### Does joint cognition change randomization?

To begin, we compared the paired sequence against both fast and slow individual sequences. The collaborative sequence was twice the length of individual sequences, and so for this analysis we randomly allocated either the first 100 or second 100 responses to each member of the pair (there were no significant differences between these response halves). Table 1 summarizes randomization performance. Owing to the statistical properties of randomness indices, we expect non-zero scores to occur even where performance is truly random. We therefore report simulated “ideal” scores to provide context for the observed values. This was achieved by sampling 2000 quasi-random 100 response sequences involving 10 alternatives, and then calculating descriptive statistics. We used an inbuilt function within a version of RGCalc [22] to run these simulations.

**Table 1:** Mean randomness scores in Experiment 1, with standard deviations in parentheses.

	Individual and paired sequences (100 responses)				True pair and composite sequences (200 responses)		
	Slow	Fast	Paired	Ideal	True pairs	Composite	Ideal
Digram Use (RNG)	.273 (.026)	.284 (.039)	.258 (.029)	.241 (.030)	.345 (.016)	.315 (.010)	.323 (.013)
Adjacency (A)	15.8 (7.53)	23.7 (8.15)	20.8 (6.35)	17.9 (4.65)	20.8 (5.50)	18.4 (1.96)	18.0 (2.76)
Immediate Repetition (RD1)	1.10 (2.25)	1.05 (2.47)	1.15 (1.19)	9.91 (2.91)	2.4 (1.88)	20.5 (3.17)	19.8 (4.41)
Redundancy (R)	1.09 (.904)	1.16 (.967)	.779 (.469)	1.98 (.950)	.519 (.335)	.501 (.346)	.983 (.455)

Note. Slow = individual sequence at a slow response rate. Fast = individual sequence at a fast rate. Paired = turn taking by two individuals. The fourth and seventh columns represent "ideal" values from computer-based simulation (the same values are provided in Table 3) and the sixth represents "composite" values. See text for description of performance metrics.

Digram Use (or more precisely “RNG” score) is a widely used metric of stereotyped sequencing in random generation [23]. Digram Use identifies preferential selection of particular two-item permutations among all responses (i.e., the score increases as digram combinations are used repeatedly). Formally,

$$\text{RNG}=\frac{\Sigma n_{ij}\;\text{log}\;n_{ij}}{\Sigma n_{i}\;\text{log}\;n_{i}}$$

where *n*_*ij*_ is the frequency count from each cell in a digram matrix, and *n*_*i*_ represents the frequency of occurrence of alternative *i*. The Digram Use scores differed across conditions, *F*(2, 78) = 7.48, *p* = .001, *η*^*2*^ = .161; the paired sequence was more random than individual-fast, *t*(39) = 3.61, *p* = .001, *η*^*2*^ = .250, and individual-slow conditions, *t*(39) = 2.58, *p* = .014, *η*^*2*^ = .145.

A related but more specific metric of stereotyped sequencing is the Adjacency (“A”) score, which reflects the frequency count of neighboring choices such as when 6 follows 5 or 2 follows 3. Adjacency analysis also revealed that performance differed across conditions, *F*(2, 78) = 23.5, *p* < .001, *η*^*2*^ = .376; the paired sequence was more random than the individual-fast condition, *t*(39) = 2.34, *p* = .024, *η*^*2*^ = .123, but less random than the individual-slow condition, *t*(39) = 4.19, *p* < .001, *η*^*2*^ = .310. The prevalence of numerically neighboring values in the paired condition, compared to the individual condition at the same overall speed, indicates that participants are prone to “adopt” responses that form continuations of their partner’s choice (e.g., she said 3, and he then said 4). Thus, the results provide evidence for sequence contagion within the pair. Moreover group performance falls short of what individuals achieve when operating at the same speed; participants are better at avoiding adjacent responses to their own choices than to their partners.

To examine response repetition, we used Immediate Repetitions (RD1), which is a mean frequency count for the same response choice twice in succession, such as 7 followed by 7. As is very commonly found, Immediate Repetitions are substantially lower than one would expect in a truly random sequence (see the “ideal” values from simulation of random selection of values). An ANOVA suggested no significant differences between conditions, *F*<1, *η*^*2*^ = .003.

However, data are highly skewed because of the rarity of repetitions. We therefore also used a Friedman’s analysis of ranks or Wilcoxon paired test throughout as a non-parametric alternative. The inferential outcome was identical for all tests in Experiments 1, 2 and 3, except for the present case. Here, a contrast revealed more repetitions in the paired condition, χ^2^(N = 40) = 10.22, p = .006, Kendall’s W = .128, an effect focused on the contrast between paired and individual-fast condition, *z* = 2.06, p = .040, while paired and individual-slow comparison was not significant, *z* = 1.58, p = .115. Notwithstanding it is evident from Table 1 that all experimental conditions cluster together and are quite different from ideal values. Since non-parametric tests elsewhere converge with the parametric, we subsequently report parametric test values only, for consistency with analysis on other indices.

Finally, we used the Redundancy (“R”) index to explore the evenness of the response frequency distribution. This measure of information redundancy assesses whether participants generated each possible alternative as often as others. Formally,

$$R=100\;\cdot \left(1-\frac{H_{single}}{H_{max}}\right)$$

where

$$H_{single}={\text{log}}_{2}n-\frac{1}{n}\left(\sum n_{i}{\text{log}}_{2}n_{i}\right)$$

and

$$H_{max}={\text{log}}_{2}a$$

where *n* is the sequence length, *n*_*i*_ is the number of occurrences of the *i*th response alternative and *a* is the number of different alternatives. The Redundancy scores differed between conditions, *F*(2, 78) = 4.50, *p* = .028, *η*^*2*^ = .103. More specifically, the numbers generated in the paired sequence were more evenly distributed than in the individual-fast, *t*(39) = 2.34, *p* = .024, *η*^*2*^ = .123, and individual-slow conditions, *t*(39) = 2.07, *p* = .045, *η*^*2*^ = .099.

#### Is joint cognition performance related to social desirability?

We also examined performance with respect to social desirability. There is no obvious reason to expect individual performance to be associated with social desirability (indeed, all *r*s(38) < .174, *p*s>.285). Importantly, there were no significant correlations between social desirability and paired performance either, *r*s(38) < .186, *p*s>.252. Similarly there was also no significant correlations between paired randomness and an aggregated social desirability score from both members of the pair, *r*s(18) < .395, *p*s>.084. These results suggest that joint cognition success may not be linked to this specific dimension of social orientation.

#### What does the individual contribute to paired performance?

**Fig 1:**
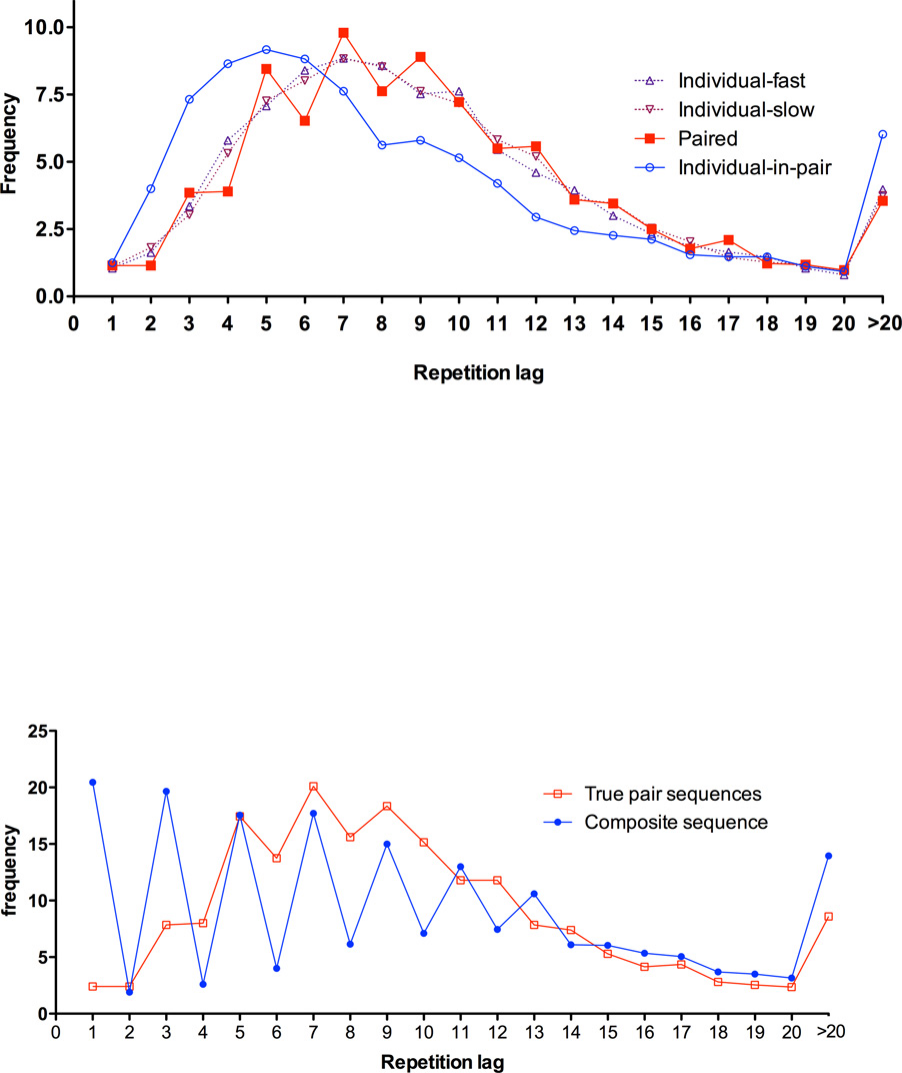
Repetition distance frequencies, the response gaps between each value and its subsequent re-appearance, in Experiment 1. Upper panel (A) represents individual conditions (slow and fast), the paired sequence and the profile for individual choices within the paired condition. All based on 100-item sequences. Lower panel (B) describes the 200 responses in the joint cognition condition and a composite sequence formed by merging responses made by each partner in their own individual sequences.

Next, we examine in more detail how individual responses come to shape the combined product. An informative visual description of randomization can be obtained from noting the spacing between repetitions of response values. Fig 1A reports these distances, or lags, between response repetitions in both the individual and paired sequences. Typical human sequences show few short-lag repeats, with frequencies rising to a peak with about six intervening responses. Paired sequences show a similar performance profile to both slow and fast individual sequences, leading to superposition of data. This finding serves to emphasize what was established above, that there is a roughly similar response repetition behavior in the paired and individual conditions.

Data from the individual-in-pair condition–considering only the responses made by a particular participant as part of a paired sequence–also shown in Fig 1A. This illustrates that this broad comparability is obtained through a marked shift in what the individual does within the dyad. Participants repeat their own choices more quickly when they take turns in generating a sequence, compared to when they work alone. Thus, whilst there was no significant difference in immediate repetition frequency between the slow condition and individual’s responses in the paired sequence, *t*(39) = .41, *p* = .683, *η*^*2*^ = .004, repetitions with one and two intervening responses occurred significantly more often in sequences produced by individuals as part of the paired output, *t*(39) = 5.01, *p* < .001, *η*^*2*^ = .392, and *t*(39) = 8.21, *p* < .001, *η*^*2*^ = .633, respectively. This result emphasizes the embodiment of the partner’s responses into the choices made by individuals.

#### Reactivity in joint cognition

Drawing upon responses from the individual (slow) condition, we constructed a composite group sequence. We interleaved individual responses, such that where each pair member A and B produced respectively as individual sequences “a_1_, a_2_, a_3_…” and “b_1_, b_2_, b_3_…”, we forged the composite sequence “a_1_, b_1_, a_2_, b_2_, a_3_, b_3_…” By creating such a baseline–a paired sequence derived from participants who are genuinely ignorant of their partner’s contribution–we can investigate the cognitive dynamics of real turn-taking. These artificial composite sequences can be compared with true pair sequences, where individuals do know what their partner has produced. We can therefore assess whether pairs do react to what their partner has said. Table 1 (right hand panel) describes performance using the performance metrics already outlined, here using the full 200 sequence sets. We again report simulated “ideal” values based on the relevant parameters (i.e., longer sequences).

Analysis of stereotypy as indexed by Digram Use demonstrates clearly that true pair sequences were *less* random than the composite sequences, *t*(19) = 7.17, *p* < .001, *η*^*2*^ = .730. The difference for the Adjacency metric was in the same direction but only marginally significant, *t*(19) = 1.84, *p* = .081, *η*^*2*^ = .151. The frequency of Immediate Repetitions in paired sequences was much more rare and less random than with composite sequences, *t*(19) = 20.12, *p* < .001, *η*^*2*^ = .955. Indeed, Immediate Repetition frequency in composite sequences was not different from mean values expected from true random sequences, *t*(19) = 1.19, *η*^*2*^ = .069.

Fig 1B contrasts repetition distances for paired and composite sequences. The clear zig-zag pattern in the composite condition reflects (1) serial dependence with respect to one’s own sequence history evident on even repetition lag values (participants know what they have said themselves, and this matters for the choices made), together with (2) serial independence of the “partner” for odd repetition lag values (since by design they cannot know what the “partner” has said).

Analysis on the evenness of response choices as measured by Redundancy scores indicated no difference between paired and composite sequences, *t*(19) = .281, *p* = .782, *η*^*2*^ = .004.

### Discussion

We have demonstrated some specific, albeit modest, benefits when pairs of individuals generate random sequences. Joint cognition sequences showed an even distribution of response choices and were less stereotyped in general than individual sequences, meaning that there was a reduced reliance on all digram combinations. This finding supports our prediction that individuals are partly influenced by idiosyncratic stereotyped relationships. Thus, joint performance on Digram Use allows for some improvement in output quality because each participant reacts differently to the same prompt, effectively redistributing response combinations.

In contrast, Adjacency responses remained prevalent in the joint cognition environment. This reflects how the Adjacency measure reflects population-wide associations, and here pairs do not do as well as individuals working at the same overall rate. This in turn confirms the strong response contagion effect in joint cognition; individuals are strongly influenced by their partner’s contribution.

In one important respect, performance does not change markedly when pairs produce random sequences. Although paired responses led to more repetitions–when using non-parametric analysis–repetitions remained amongst the least preferred continuation choices. Thus, the data provide evidence against the hypothesis that repetition avoidance in random generation derives from the automatic suppression of *self-generated* responses. The present data instead suggest that Immediate Repetitions are strongly dis-preferred regardless of the source of the first selection. Moreover, it is apparent that response activation levels do not recover until several further responses have been made. Indeed, this further underscores the response contagion effect; we avoid repeating our partner’s choices as much as we avoid repeating our own.

## Experiment 2

In Experiment 1, we studied performance of two naïve participants who have been brought together in an experimental session. Both are trying to produce random sequences, though we know each is likely to fall well short of this. Such data are complemented by studying a related scenario, in which one half of the pair does make random choices. Consider the situation when a participant is part of dyad, and his or her partner is calling random responses, but those that are unrelated to their own. In Experiment 2, we took advantage of an inevitable aspect of experimental recruitment; sometimes only one participant signed (or turned) up for an experimental timeslot. This actually provided a unique opportunity, as follows. We paired the participant with an experimenter, who gave their responses using a pre-prepared, quasi-random response set, with participants in full knowledge of this constraint (i.e., this was not a confederate study). This design means that we can examine whether group phenomena (such as sequence contagion) occur only with participatory situations that are (conceptually) symmetrical, that is, situations where each member of the pair is trying to make the combined sequence fit the task requirements. Is an individual freed from the constraints of reactivity when they know that their partner is not interacting with them?

### Method

#### Participants

Sixteen participants took part from the same participant pools as Experiment 1; failure to record a complete response set led to one participant being subsequently excluded. Ages were not recorded. With data collected alongside those for Experiment 1, the same ethical principles, and procedure, applied here also. Data were collected in 2007.

#### Procedure

In the joint cognition condition, it was explained to participants that they would be taking turns to generate a sequence alongside an experimenter. That experimenter would read from a pre-prepared computer-generated response set, which was written down on paper. The experimenter’s to-be-selected responses were not visible to the participant, though the response sheet was. The same response set was used for all participants, and values were quasi-random in that they were generated by a computer with the constraint that each response alternative was selected equally (so as to avoid the subjective judgment of inequality). In all other respects, the methods followed Experiment 1.

### Results

To begin, we demonstrate the equivalence across the two datasets (Experiments 1 and 2) in participants’ solo random generation performance. None of the four measures of sequence structure described so far (Digram Use, Adjacency, Immediate Repetition and Redundancy) showed a significant difference between the current participants and those in Experiment 1, either at fast or slow pace, all *t*s<1.18, *p*s>.241, *η*^*2*^ < .026. This finding confirms that, at the level of solo performance, there was nothing unusual about what this sample did in generating random numbers.

A central motivation for this study is to investigate whether participants show effects of joint cognition even though they are working alongside a non-responsive (i.e., non-interactive) person. It is not appropriate to run exactly the same suite of paired analyses as used in Experiment 1 because of the known properties of the Experimenter’s responses. For example, the group sequence will be more random than individual sequences merely by virtue of incorporating quasi-random responses to the conjoint set. Nonetheless, several key issues can be addressed.

**Fig 2:**
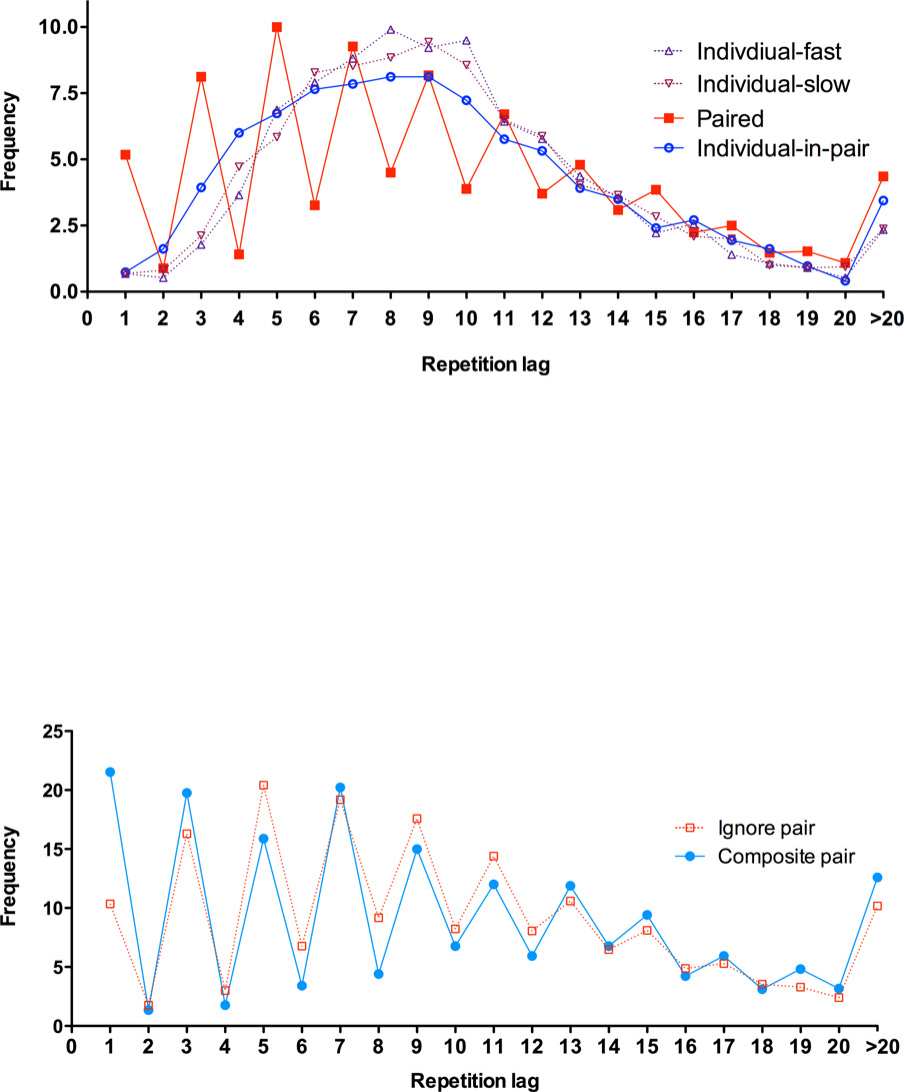
Repetition distance frequencies in Experiment 2. Data plots represent the individual conditions (slow and fast) and the individual contribution to the group sequence. All based on 100 item sequences.

In particular, we analyse the individual-in-pair responses within the paired condition, to compare participants working with another true participant (participant-participant [P-P] pairs in Experiment 1) against participants sharing the sequence with pre-selected fixed choices (participant-experimenter [P-E] pairs in Experiment 2). As summarized in Table 2, all four measures of sequence quality were statistically equivalent, all *t*s<1.49, *p*s>.146, *η*^*2*^ < .041. Moreover, the repetition lag profile in Experiment 2 (illustrated in Fig 2) was also similar to that in Experiment 1 (Fig 1A). Thus, participants’ contribution to group performance is broadly similar regardless of the opportunity for their partner to react to them.

**Table 2:** Mean randomness scores for Participant-Participant pairs (P-P: Experiment 1) and Participant-Experimenter pairs (P-E: Experiment 2). Based on individual-in-pair contributions of 100 item sequences. Standard deviations in parentheses.

	P-P pairs	P-E pairs
Digram Use (RNG)	.283 (.032)	.268 (.039)
Adjacency (A)	20.2 (5.08)	20.8 (6.37)
Immediate Repetition (RD1)	1.25 (1.60)	2.27 (3.03)
Redundancy (R)	2.38 (1.38)	2.35 (2.12)

Note. See text for description of performance metrics.

Finally, we consider the possibility that participants might notice that when paired with a pre-set sequence, the computer’s responses repeat what they have articulated (even while participants do not repeat what the computer does). We examined the number of immediate repeats in participants’ individual slow and fast conditions, as a function of whether data were produced before or after the P-E pair condition. Participants made immediate repeats more frequently after the P-E pair condition (*M* = 4.29, *SD* = 7.52) than before (*M* = 1.11, *SD* = 1.53), although with the small sample size and large variability in the former condition, this difference was not significant, *t*(13) = 1.25, *p* = .234, *η*^*2*^ = .107. We suggest that a more targeted examination of this question could possibly show that participants adapt their normative model of random sequences in light of exposure to and interaction with a random sequence.

### Discussion

The study offers a procedurally simple extension to the concept of joint cognition, isolating the contribution of each person’s cognitions by having just one true participant, and showing that group dynamics can occur regardless of whether a participant encounters randomness in their partner’s sequences. This study does not attempt to address, for example, whether the presence of a human in the dyad is critical, nor the impact of a partner’s status (there was no pretense that the Experimenter was another participant). Nonetheless, participants again modify their individual choices to make the paired sequence the unit of analysis; as a result, their performance is remarkably similar in the current artificial pairing, as for the true pairing in Experiment 1. Participants are not greatly affected by having a partner who is much more random than they are.

## Experiment 3

The data presented so far suggest that cognitive contagion is strong in the group setting, happening even when an individual shares responses with a computer generated list (spoken by an experimenter). However, the data thus far cannot arbitrate as to whether group phenomena can be affected by the intention to cooperate. Since task instructions note that performance will be assessed in terms of the quality of the combined sequence, cooperation is, implicitly, built into the design. Yet, we have seen that cooperation may not help, and in some cases leads to sub-optimal performance (i.e., productivity loss).

In Experiment 3, we examine the counterintuitive possibility that performance might be facilitated by instructions to minimize cooperation (or to put the question another way, what are the key attributes of joint cognition without collaboration?). More specifically, we ask the following question: Can each member of the pair ignore what their partner is doing, and how does this affect performance?

In this study, we use a sample of Japanese participants. We use the data to check both the effect of task instructions with respect to cooperation but also the cultural replicability of the phenomena observed so far.

### Method

#### Participants

Sixty-six (Kyoto University) undergraduates and postgraduates (between 18 and 29 years of age) forming 33 pairs, took part and received 500 yen worth of book coupons. Approximately half of the pairs (16 pairs) were randomly assigned to the *cooperate* condition and the other half (17 pairs) to the *ignore* condition. Data were collected between 2008 and 2009.

There was no local ethics committee established at the time of running this experiment, from whom approval could be obtained. However, this study was conducted in accordance with the Declaration of Helsinki and the Ethical Principles of the Japanese Psychological Association (JPA) and the APA. Indeed, the experimental protocol was basically the same those reported above, which had been completed at the time and for which approval had been obtained. Before participation, all participants read and signed a consent form, which stated their right to withdraw the experiment at any time and for any reason. At the start of data processing, responses were made anonymous, and could be connected to identifying information only by a participant code accessible on the consent form, and which was kept separate.

#### Procedure

All participants in this experiment performed three random generation tasks; two were performed individually (fast and slow conditions) and one as a pair. Procedures followed previous experiments, except that (a) we did not administer the Social Desirability Questionnaire, and (b) we manipulated the task instructions for some pairs.

The central feature of this experiment was that, when generating random sequences in pairs, the participants were given different instructions. In the *cooperate* condition (16 pairs), participants were instructed to make their combined sequence as random as possible. This instruction is essentially the same as used in Experiment 1 except that it was provided in Japanese. In contrast, in the *ignore* condition (17 pairs), participants were explicitly instructed to focus only on making their own responses random (and thus ignore their partner’s choices).

### Results

To simplify presentation we use the same performance metrics to assess the randomness of responses. First, we confirm that data from the cooperate condition are aligned with those already reported from a different participant sample. Second, we show that participants in both instruction conditions generate similar random sequences when they act as individuals. Third, we contrast the impact of different instructions for group performance and show that these are different in some key respects.

#### Analyses for the data from the cooperative group: a replication

This study recruited participants from a different country and culture. So we first sought to confirm that data from Experiment 3 (using a Japanese sample) successfully replicated the main outcomes we reported earlier in Experiment 1 (using a US and UK sample). We describe this replication analysis in detail in the Appendix, which indeed allows us to conclude that in all major respects, performance is comparable.

#### Individual performance as a function of task instruction

In this section, we use the relevant data to establish the comparability of the individual (solo) performance for the two instruction groups. Table 3 summarizes the slow and fast individual performance from the cooperate and ignore groups and again reports simulated “ideal” scores for convenience. We conducted a two-way ANOVA with generation pace (slow vs. fast) as a within-participant factor and instruction (cooperative vs. ignore) as a between-participants factor for each of main indices of randomization quality (Digram Use, Adjacency, Immediate Repetition, and Redundancy).

**Table 3:** Mean randomness scores in Experiment 3, with standard deviations in parentheses.

	Individual and paired sequences (100 responses)				True pair and composite sequences (200 responses)		
	Slow	Fast	Paired	Ideal	True pairs	Composite	Ideal
Cooperate Group							
Digram Use (RNG)	.273 (.026)	.283 (.033)	.252 (.024)	.241 (.030)	.348 (.013)	.316 (.013)	.323 (.013)
Adjacency (A)	15.2 (6.96)	20.0 (5.76)	18.5 (4.27)	17.9 (4.65)	18.4 (3.22)	18.0 (2.79)	18.0 (2.76)
Immediate Repetition (RD1)	.688 (1.29)	.688 (1.01)	.750 (1.15)	9.91 (2.91)	1.5 (1.66)	20.8 (3.97)	19.8 (4.41)
Redundancy (R)	.889 (1.15)	.941 (1.32)	.648 (.393)	1.98 (.950)	.456 (.319)	.371 (.300)	.983 (.455)
Ignore Group							
Digram Use (RNG)	.261 (.025)	.273 (.030)	.237 (.024)	-	.321 (.011)	.319 (.014)	-
Adjacency (A)	17.2 (5.30)	20.4 (7.92)	19.8 (3.37)	-	20.0 (2.49)	16.6 (2.28)	-
Immediate Repetition (RD1)	.706 (1.54)	.676 (1.41)	5.18 (2.09)	-	10.4 (3.46)	21.5 (4.43)	-
Redundancy (R)	.628 (.370)	.689 (.425)	.674 (.523)	-	.374 (.233)	.352 (.225)	-

Note. Slow = individual sequence at a slow response rate. Fast = individual sequence at a fast rate. Paired = turn taking by two individuals. The fourth and seventh columns represent "ideal" values from computer-based simulation (the same values are provided in Table 1) and the sixth represents "composite" values. See text for description of performance metrics.

For Digram Use, as is almost universally found, the fast condition led to less random output than the slow condition, *F* (1, 64) = 4.08, *p* = .048, *η*^*2*^ = .063. We also found that for individual performance, the cooperate group were less random than the ignore group, *F* (1, 64) = 5.26, *p* = .025, *η*^*2*^ = .078, which may be attributable at least in part to a carry-over effect from the counterbalancing of conditions. (A full analyses of and partitioning of this effect is available from the authors). There was no significant interaction between the two factors, *F* (1, 64) < 1, *η*^*2*^ = .001.

Adjacency showed significantly less random output for the fast than the slow condition, *F* (1, 64) = 32.17, *p* < .001, *η*^*2*^ = .329, but no effect of instruction, *F* < 1, *η*^*2*^ = .010, or interaction, *F* (1, 64) = 1.47, *p* = .230, *η*^*2*^ = .022. There were no significant main effects or interactions for Immediate Repetition frequencies and Redundancy, all *Fs* (1, 64) < 1.40, *p*>.248, *η*^*2*^ < .021.

In addition to confirming the well-established finding that some randomness measures (Digram Use and Adjacency) are affected by generation speed, these results also confirm (not surprisingly) that the two instruction groups were generally comparable to each other in their individual random generation performance.

#### Group performance as a function of task instruction

The main focus of this experiment, however, is how the pairs’ random generation performance is affected by the instructions (cooperate vs. ignore). Table 3 compares true pair and composite pair performance for the four indices of randomness alongside simulated or “ideal” performance scores.

We conducted a two-way ANOVA with pair type (true pairs vs. composite) as a within-participant factor and instruction (cooperate vs. ignore) as a between-participants factor for each measure. With respect to Digram Use, the ignore group were more random than the cooperate group, *F* (1, 31) = 14.61, *p* = .001, *η*^*2*^ = .320, and the composite pairs were more random than the true pairs, *F* (1, 31) = 22.50, *p* < .001, *η*^*2*^ = .323. There was a significant interaction between the two factors, *F* (1, 31) = 16.24, *p <* .001, *η*^*2*^ = .234. While the ignore and cooperate groups did not differ significantly for the composite pairs, *F* (1, 62) < 1, *η*^*2*^ < .001, the ignore group showed more random sequences than the cooperate group for the true pairs, *F* (1, 62) = 30.85, *p* < .001, *η*^*2*^ = .332.

Adjacency showed a main effect of pair type, indicating more random sequences for the composite pairs than the true pairs, *F* (1, 31) = 7.13, *p* < .001, *η*^*2*^ = .168, but no effect of instruction, *F* (1, 31) < 1, *η*^*2*^ < .001. The interaction between the two factors was marginally significant, *F* (1, 31) = 4.10, *p* = .052, *η*^*2*^ = .098. For Immediate Repetitions, the ignore group showed more random output than the cooperate group, *F* (1, 31) = 33.66, *p* < .001, *η*^*2*^ = .520, and the composite pairs were more random than the true pairs, *F* (1, 31) = 243.89, *p* < .001, *η*^*2*^ = .834. Furthermore, there was a significant interaction between the two factors, *F* (1, 31) = 17.37, *p* < .001, *η*^*2*^ = .106. There was a simple main effect for the true pairs with better performance under ignore instructions, *F* (1, 62) = 47.99, *p* < .001, *η*^*2*^ = .435, but no significant effect for the composite pairs, *F* (1, 62) < 1, *η*^*2*^ = .003. There were no significant main effects or interactions for Redundancy, all *Fs* (1, 31) < 1.10, *p*>.312, *η*^*2*^ < .032.

**Fig 3:**
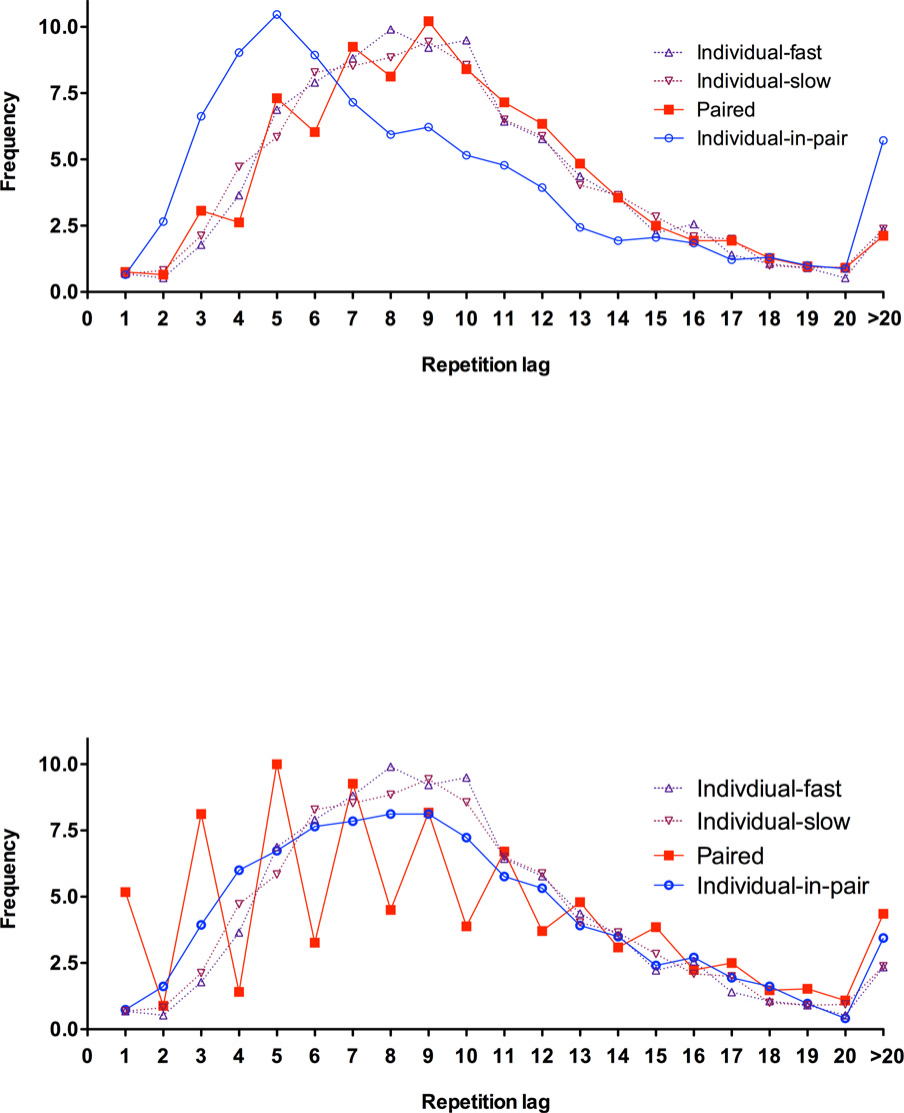
Repetition distance frequencies in Experiment 3. Data plots represent individual conditions (slow and fast), the paired sequence and the profile for individual choices within the paired condition. All based on 100-item sequences. Upper panel (A): performance in the Cooperate group. Lower panel (B): performance in the Ignore group.

Fig 3 reports the distances or lags between response repetitions in the individual and paired sequences for the cooperate condition (3A) and the ignore condition (3B). Fig 3A emphasizes the comparability of the current data with those reported in Experiment 1 (see Fig 1). Fig 3B emphasizes how under ignore instruction, participants alter their decisions. Performance of individuals in pairs much more closely approximates the individual performance–there is no longer a leftward shift in the frequency peak. In addition, the paired condition shows a much more obvious sawtooth pattern, with a disconnection between self-generated and other-generated choices.

**Fig 4:**
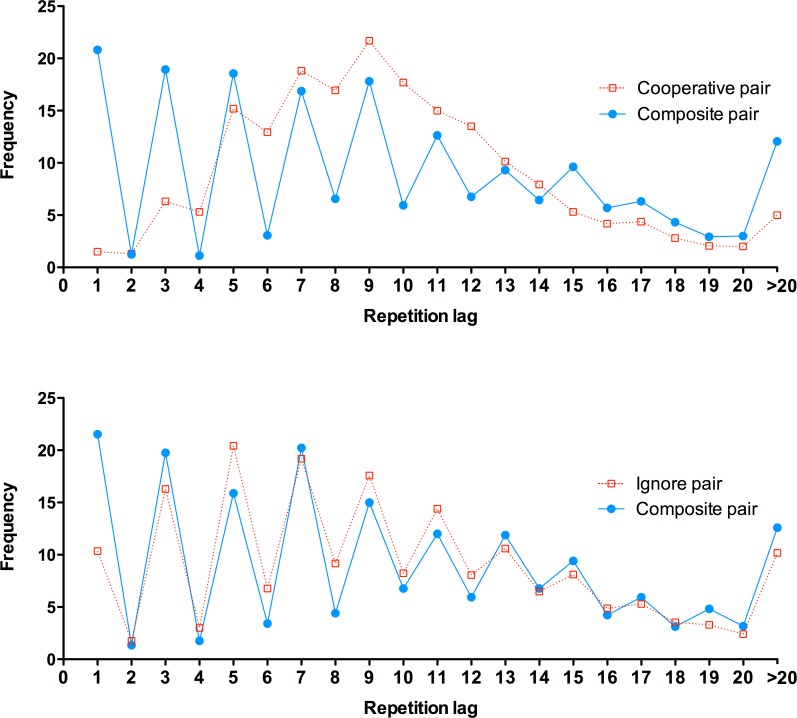
Repetition distance frequencies for the 200 responses in the joint cognition condition and a composite sequence formed by merging responses made by each partner in their own individual sequences, from Experiment 3. Upper panel (A): data from the Cooperate Group. Lower panel (B): data from the Ignore Group.

Repetition lags are explored further in Fig 4. Performance of the pairs is compared against composite sequences that come from the merging of each person’s individual production sets. Fig 4A describes the cooperate condition and 4B the ignore condition. Under instructions to cooperate, data (4A) strongly confirm the pattern previously described in Fig 2. When asked to ignore their partner, the paired performance (4B) looks very different. The repetition behavior of the ignore pairs is now much more similar to composite data where participants cannot know their co-contributors selection–(because they are produced independently). In other words, participants better approximate random (i.e., independent) choice selection with respect to their partner, whilst there remains a strong dependency with respect to one own choices. Immediate Repetitions are the data point with the greatest discrepancy between formats.

### Discussion

This experiment addresses several key issues. First, with respect to the impact of group dynamics and intention to cooperate, we introduced a request to ignore the contribution of the dyadic partner. This led broadly to performance improvement. That is, participants avoided some of the negative consequences from entrainment (or thought contagion) that is evident in the more collaborative situation. Ironically, therefore, when individuals in a pair try to not collaborate, their conjoint performance can surpass that of pairs who are trying to make the combined performance as good as possible. Moreover, this finding is entirely consistent with the analysis from composite pairs, showing that truly being ignorant of what another person might say is advantageous to randomization quality. It is also consistent with Experiment 2 in which a participant took turns to respond with another who read from a pre-specified list, where we also obtained evidence of entrainment when the combined output was emphasized as the target performance.

Thus we conclude that at least some of the costs of joint cognition on performance can be ameliorated. At the same time, the ignore condition does not generate ideal performance; group formation continues to carry residual performance costs. This is particularly evident when one compares the mean performance achieved in the ignore group in Experiment 3 with the idealized simulation data in Table 1. Ignoring your partner is a better strategy than cooperating with them, but neither makes you random on most performance dimensions.

Second, we note that data replicate the findings from the Experiment 1, in all major respects–see Appendix for full details. This is noteworthy for at least two reasons. First, the data point to the robustness of the effects already reported. Second, insofar as the current experiment involves participants from a different sample (involving a less individualistic cultural ethic), the data lay the groundwork for considering the impact of instructional emphasis; the replication provides confidence in the baseline condition of cooperative intent.

## General Discussion

In three experiments, we have investigated how group random generation performance compares with individual performance, and established how the nature of the relationship within the group can matter. We orient our discussion about how these studies inform a better understanding of random generation and joint cognition in the context of a set of six key findings. We specify and discuss each in turn.

Our first key finding is that there are both costs and benefits from joint cognition. It is clear that even when one finds benefits from working alongside another person on a random generation task, such benefits not lead to anything like a simple halving of performance constraints. Random sequence generation is not resource-limited in any simple, linear way (despite its common deployment as a secondary task depriving the randomizer of executive resources). Instead, what emerges is actually a much more subtle and informative account of task sensitivity, with the consequences of group performance varying according to the dimension of analysis and the configuration of the task.

We suggest that this finding underlines the conceptual value of using a complex task like random generation to explore joint cognition. The current paradigm, when used with appropriate and focused methods of analysis, allows for a nuanced analysis of performance, rather than a potentially oversimplified global message such as “joint cognition is superior / inferior” to individual performance. Indeed, whilst theoretical and conceptual predictions have led us to frame specific analytic outcomes in terms of whether or not paired or individual sequences better approximate characteristics typical of truly random sequences, these comparisons are relative. That is, a change in the detectable regularity in a sequence could arise from different causes; we note past studies sometimes indicate increased task load can lead to “improved” scores (e.g. [24]). In some other circumstances analysis could be framed simply and neutrally as changes to a performance metric, that is, without a relative statement.

### Implications for models of random generation

Our second key finding is that strong repetition avoidance is found in group sequences as well as individual sequences. This represents a major contribution of the present data with respect to cognitive models of random generation. Although random generation research (at least with respect to non-binary environments) often focuses on the stereotyped nature of response sequence chains, the avoidance of immediate and short-lag repetitions of a response is perhaps the most noticeable and persistent feature of choice behavior in random generation datasets. Whilst the repetition of a just-given item should be as common as any other single option, actually many 100-response sequences contain no immediate repetitions at all. Repetition avoidance has been interpreted in terms of response suppression, the automatic inhibition of just articulated responses. However, repetition avoidance is highly prevalent in much group output, indicating that a choice is effectively discounted once produced, regardless of who produced it.

More generally, with respect to the concept of executive functioning, the results of the current study further emphasize the complex and multidimensional nature of this concept; simple resource accounts of executive functioning fall short of explaining the properties of joint cognition. There are dissociations in performance across measurement scales and between individual and paired performance. In other words, different dimensions of control fractionate at a psychological level, and this is on the basis of a single paradigm only they are undoubtedly other skills captured by different tasks. In addition to the self-monitoring requirements inherent in at least some executive functioning situations, we suggest that other-monitoring may also feature in cooperative multi-person contexts, modulated by a reactivity effect whereby partner’s responses may be represented as equivalent to one’s own.

### Implications for joint cognition research

Our third key finding is that participants are sensitive to the group context and setting of the joint cognition task. In other words, participants change their behavior in a group setting. Thus, in all experiments, and especially Experiments 1 and 2, individuals’ repetition response profile is different in dyads, as self-repetitions occur more quickly in the former case. Moreover, individuals choose numbers adjacent to the value of that generated by their dyad partner and in addition participants’ group performance is comparable whether one provides real or artificial partners (Experiment 2). Indeed, we also showed that in some respects sequence quality is enhanced if participants are able to ignore the choices made by their partner. This was apparent in the creation of composite sequences and through the specific instructions in Experiment 3, and underscores the impact of another person under a conventional setting. Interestingly, a recent study of joint task switching published after our submission [25] has also found that the impact of a shared task can be distinguished from the impact of task manipulations.

Our fourth key finding is an extension of the third, that group performance is different. It is that joint cognition can produce intellectual contagion to the partners’ sequences (which occurs for response associations, response repetitions, and item usage frequencies). Such reactivity or social contagion to the responses of others is important, theoretically and practically. There is a sense in which the pairs began “thinking on the same wavelength”, and potentially this might form a demonstration of the cognitive analogue of social conformity.

This conclusion resonates nicely with findings from the “Wisdom of Crowds” effect–the phenomenon whereby the composite of many individual estimations can be highly accurate even when individual responses are quite inaccurate. [26] have shown that when individuals have information about others’ choices, the benefit or gain from aggregating group responses can be reduced. To the extent that social contact can produce cognitive contagion, individual choices no longer become independent, and this is an import prerequisite for the Wisdom of Crowds effect.

Whilst in other experimental situations, one can observe the chameleon effect [27], that is a non-conscious mimicry of one’s interaction partner, here we do not observe responses that repeat the partner’s, but instead follow on from them. In other words, the contagion is a projection of the partner’s adjacent sequence (see [28], for a similar psycholinguistic effect in dialogue production).

This contagion effect has various implications for joint cognition paradigms more widely, consistent with the conclusion that collaboration affords opportunities for both productivity loss and productivity gain [29]. In circumstances where a high concordance of ideas is positive, joint cognition may be beneficial. Yet, by restricting the diversity of thought, joint cognition may also limit the opportunity for radically new decision patterns. The absence of emergent performance properties in the present paradigm resonates with other situations such as collaborative recall, where there is often a failure to produce information inaccessible to both individuals [15]. However, executive function is assessed here along multiple dimensions. A clear message from the current work is that joint cognition outcomes are not all-or-none in terms of costs and benefits. There is evidence for both, which enriches the appreciation of the multiple processes involved.

Our fifth key finding is that group effects can arise even without both the partners being active players in the social dynamic. We observed social contagion in Experiment 2 even when participants knew that their partner was behaving independently of them. The conclusion implies that cognitive reactivity, if not automatic, is strongly triggered by the social situation. This finding also speaks to the issue of group performance plans or coordination. Participants were not given the opportunity to discuss how they might optimize performance in pairs. We recognize that the absence of explicit collaborative interactions might affect behavior. Yet, we also note that in many real-world situations individuals work together without explicit consideration of collaborative roles. Our finding that joint cognition was not related to social desirability provides one constraint on how individual differences might modulate group performance, but of course it does not preclude the possibility that other measures might show a stronger mediating role.

The sixth finding we emphasise is that in Experiment 3 the instructions to ignore or neglect the partner’s contribution benefited the shared response. That is, instructions to cooperate may not benefit the quality of the group data. This may seem counterintuitive at first, but shared ownership of partner’s responses may make it hard to overcome the limitations that such alignment brings. This in turn gives a further important clue to the potential optimization of group performance in joint cognition environments.

### Concluding remarks

Frith [30] has discussed the social brain hypothesis in detail. Generalizing from the human mirror system, Frith argues that “actions are contagious”. Our data suggest that indeed it is not just actions, but thoughts too, that can have the potential for this psychologically important property. As a consequence, a socially shared cognitive task offers a rich environment to study group performance dynamics (e.g., [8,10,28] and an important complement to the study of solitary mental processes.

## Appendix: Analysis to show Experiment 3 replicates the key findings from Experiment 1

Experiment 3 allowed us to compare a cooperative version of the paired random generation task against an ignore version. The former version is explored in Experiment 1, and therefore, we analyze performance to check that the key results of this initial experiment can be replicated, despite the different participant sample. The upper part of Table 3 summarizes randomization performance in the two solo conditions (fast and slow) and the paired condition from the cooperative group alone.

Performance as assessed by Digram Use differed across conditions, *F*(2, 62) = 8.25, *p* < .001, *η*^*2*^ = .210; the paired sequence was more random than individual-fast, *t*(32) = 3.85, *p* < .001, *η*^*2*^ = .218, and individual-slow conditions, *t*(31) = 2.87, *p* = .007, *η*^*2*^ = .146. Adjacency also differed across conditions, *F*(2, 62) = 7.87, *p =* .001, *η*^*2*^ = .202; the paired sequence was less random than the individual-slow condition, *t*(31) = 2.13, *p* = .041, *η*^*2*^ = .075 while not different to the individual-fast condition, *t*(31) = 1.24, *p* = .226, *η*^*2*^ = .022. Again, the results provide evidence for sequence contagion within the pair and once again group performance falls short of what individuals achieve when working at the same speed.

There was no evidence that repetition avoidance bias changed in the joint cognition environment. Table 3 reports the mean frequency of Immediate Repetitions and as in Experiment 1, values are substantially less than one would expect in a random sequence. There were no significant differences between conditions, *F*<1, *η*^*2*^ = .005. Redundancy did not differ between conditions, *F*(2, 62) = 1.63, *p* = .205, *η*^*2*^ = .050.

Fig 3A reports the distances or lags between response repetitions in the individual and paired sequences under instructions to cooperate. Replicating Experiment 1, paired sequences show a very similar performance profile to both slow and fast individual sequences, leading to superposition of data. This figure also confirms that participants repeat their own choices more quickly when they take turns in generating a sequence compared to working alone. Whilst there was no significant difference in immediate repetition frequency between the slow condition and individual’s responses in the collaborative sequence, *t*(15) = 0.17, *p* = .865, *η*^*2*^ = .001, repetitions with one and two intervening responses occurred significantly more often in sequences produced by individuals as part of the group sequence, *t*(15) = 4.02, *p* < .001, *η*^*2*^ = .343, and *t*(15) = 7.35, *p <* .001, *η*^*2*^ = .635, respectively.

Table 3 compares four random indices between true pair and composite pair performance. Analysis of stereotypy as indexed by Digram Use demonstrates clearly that paired sequences were *less* random than the composite sequences, *t*(15) = 6.41, *p* < .001, *η*^*2*^ = .732. The effect for Adjacency was not significant, *t*(15) = 0.43, *p* = .677, *η*^*2*^ = .012, and there was less Immediate Repetition in paired than composite sequences, *t*(15) = 17.96, *p* < .001, *η*^*2*^ = .956. Indeed, immediate repetition frequency in composite sequences was not different from values expected in random sequences (see Fig 4A). Analysis on the evenness of response choices as measured by R scores indicated no difference between paired and composite sequences, *t* (15) = 1.07, *p* = .303, *η*^*2*^ = .071.

In summary, the data from the cooperative group replicated those from Experiment 1 in all major respects. In particular, pairs show less stereotypy than individuals as measured by Digram Use, but not Adjacency. This confirms the discontinuity between associates that are neighboring numbers and digram associations. Moreover, repetition was equally strong in the individual and paired condition. True pair sequences were not as random as composite pairs, and thus in this respect, combining individuals led to performance impairment.
